# Superficial Ewing Sarcoma of the Rectum: A Case Report and the Utility of Molecular Diagnostics

**DOI:** 10.1111/cup.70128

**Published:** 2026-05-07

**Authors:** Jessica L. Muldoon, Laura M. Warmke, Ahmed K. Alomari, Rachel P. Kowal

**Affiliations:** ^1^ Clinical Pathology & Laboratory Medicine Indiana University School of Medicine/Indiana University Health Indianapolis Indiana USA

## Abstract

Ewing sarcoma is an undifferentiated small round cell sarcoma that most commonly presents as a malignant bone tumor in pediatric and young adult patients. The diagnosis is typically confirmed by molecular genetic identification of a fusion protein, most commonly involving members of the FET and ETS gene families. The most frequent translocation is *EWSR1*::*FLI1* which occurs in approximately 85% of cases. Rare extraosseous cases of superficial Ewing sarcoma have been described, including one molecularly confirmed tumor involving the rectum. Herein, we report the second molecularly confirmed case of a superficial, rectal primary tumor arising in a 38‐year‐old male who initially presented with bleeding. Physical exam noted an ulcerated rectal polyp near the dentate line. Excisional biopsy of the lesion revealed a monotonous round cell malignancy involving the submucosal tissue with necrosis and marked mitotic activity. Immunohistochemical studies showed reactivity for CD99 and NKX2.2 with weak, focal expression of synaptophysin. Next‐generation sequencing revealed an *EWSR1*::*FLI* fusion, confirming the diagnosis of Ewing sarcoma. The fusion variant junction was located in exon 7 for EWSR1 and exon 6 for FLI1. Subsequent imaging revealed no evidence of metastatic disease, and re‐excision showed no residual tumor. Primary superficial Ewing sarcoma is extremely rare, and this is only the second molecularly confirmed case of rectal origin.

## 1 Introduction

Ewing sarcoma is a malignant small round blue cell tumor that most commonly arises in the long bones of patients less than 20 years old [1]. Approximately 10% of Ewing sarcomas present as an extraskeletal tumor and even more rarely as superficial lesions, including those arising in cutaneous and mucosal locations [2, 3, 4]. While immunohistochemical evaluation helps to narrow the differential, molecular evaluation confirms the diagnosis and excludes the possibility of other round cell sarcomas. Ewing sarcoma is defined by the presence of fusion proteins most frequently involving members of FET and ETS gene families. Specifically, *EWSR1*::*FLI1* is the most common gene fusion followed by *EWSR1*::*ERG* [1].

## 2 Case Report

**FIGURE 1:**
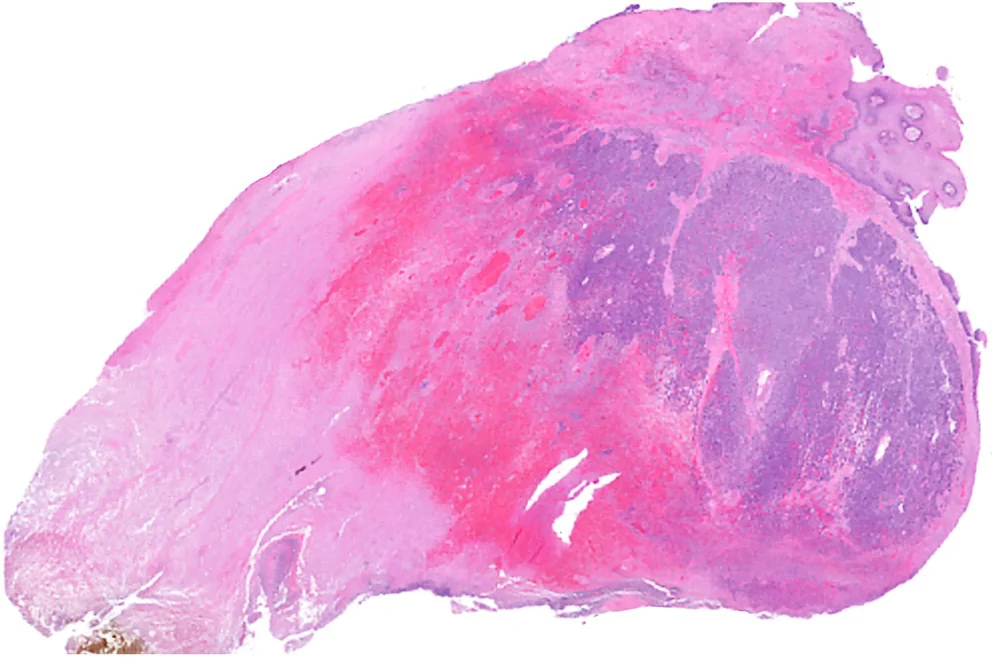
Hematoxylin & Eosin (H&E, 25×). Submucosal, polypoid lesion composed of small round blue cells with areas of necrosis and hemorrhage (left).

**FIGURE 2:**
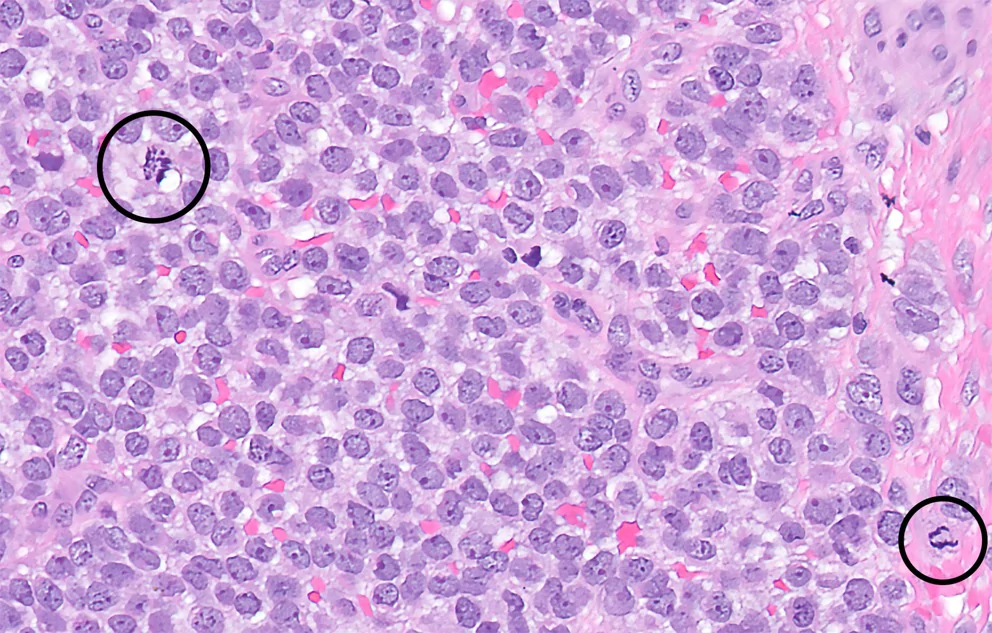
Hematoxylin & Eosin (H&E, 200×). Monotonous small round blue cell tumor with prominent nucleoli, scant clear to eosinophilic cytoplasm, and increased mitotic activity (circled).

**FIGURE 3:**
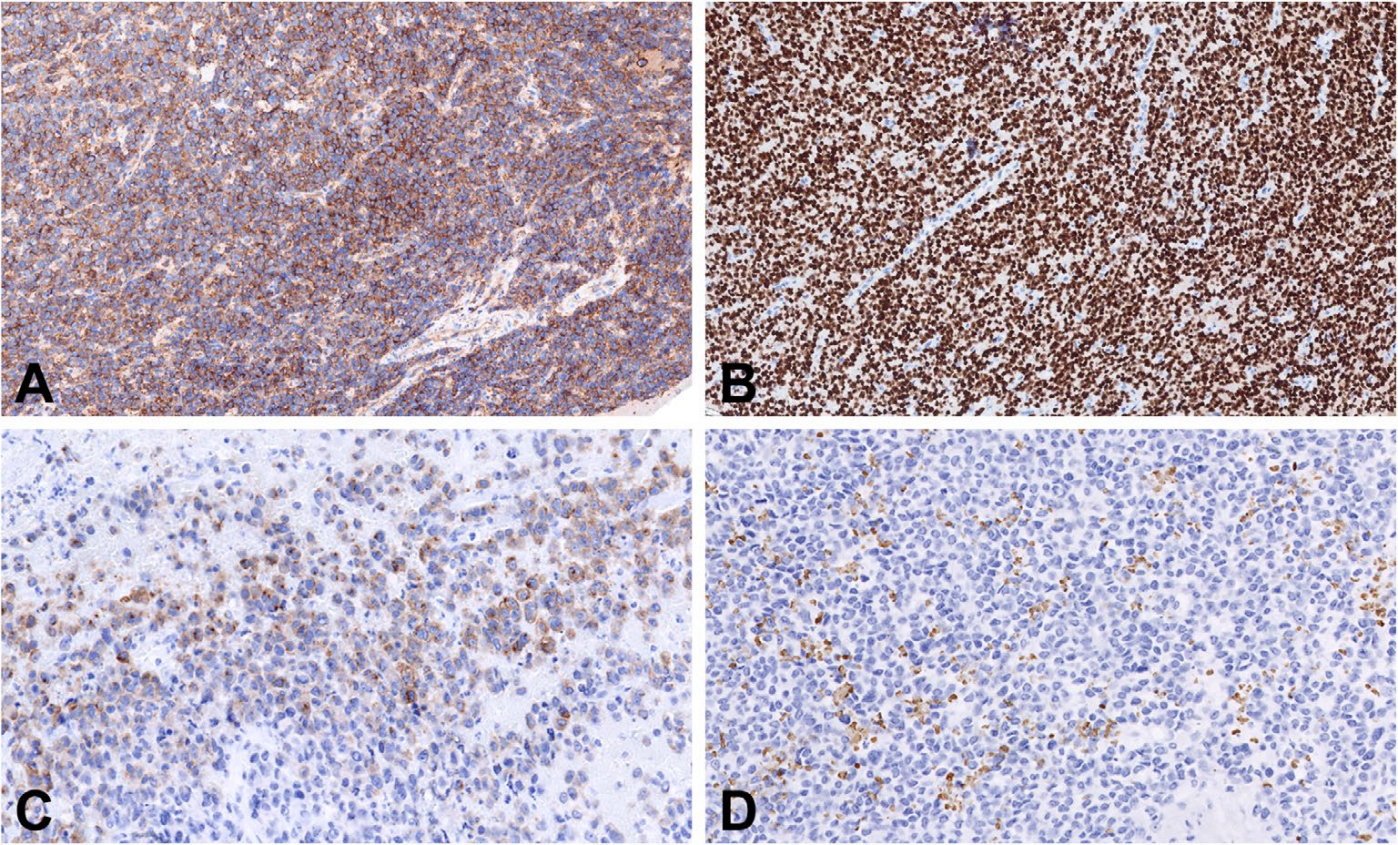
(A) CD99 (IHC, 100×). The tumor cells showed strong, diffuse staining with CD99. (B) NKX2.2 (IHC, 100×). The tumor cells showed strong, diffuse staining with NKX2.2. (C) Synaptophysin (IHC, 100×). The tumor cells demonstrate focal, weak expression of synaptophysin. (D) Chromogranin (IHC, 100×). The tumor cells demonstrate focal, weak expression of chromogranin.

**FIGURE 4:**
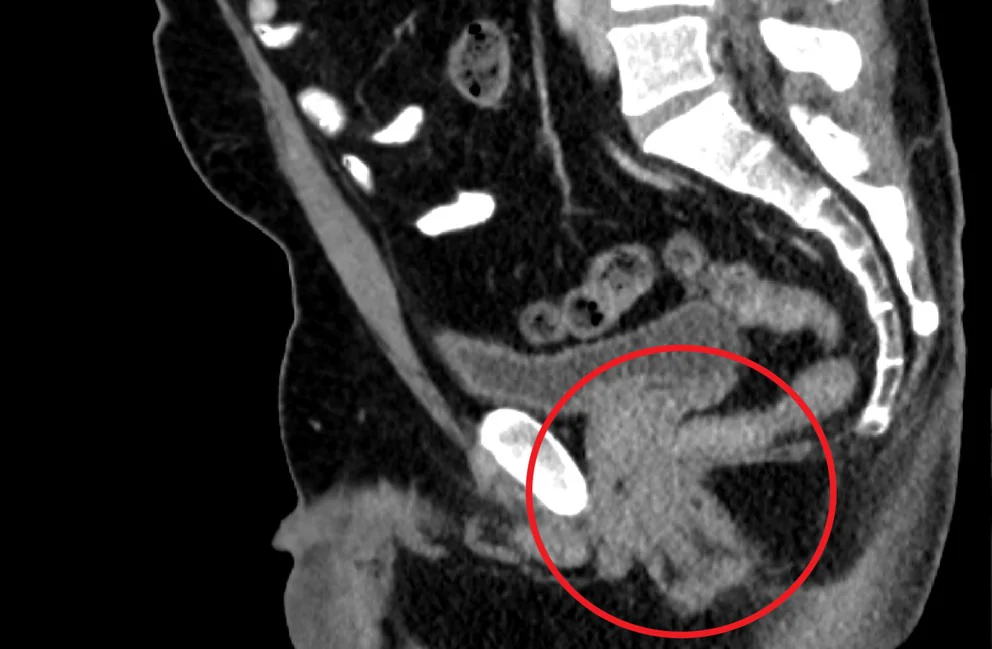
Abdominal CT scan with contrast (sagittal view) demonstrating diffuse rectal thickening (circled).

A 38‐year‐old man with a history of hypertension and hyperlipidemia presented with rectal bleeding that did not respond to over‐the‐counter treatment for presumed hemorrhoids. Physical exam noted a painful protruding and ulcerated lesion near the dentate line, suggestive of a prolapsed rectal polyp. An excisional biopsy demonstrated a poorly differentiated, round blue cell tumor involving the submucosal tissue with geographic tumor necrosis (Figure 1). The tumor was composed of monotonous, small round blue cells with prominent nucleoli and marked mitotic activity (up to 42 mitotic figures per 10 HPF) (Figure 2). Immunohistochemical stains showed reactivity for CD99 (diffuse, membranous) (Figure 3A), NKX2.2 (diffuse, nuclear) (Figure 3B) and focal, weak expression of synaptophysin (Figure 3C) and chromogranin (Figure 3D). The tumor cells were essentially negative for CK34BE12, CKAE1/AE3, CAM5.2, CK5/6, SALL4, OCT4, SOX10, S100 protein, Melan‐A, HMB45, INSM1, CD56, CD45, CD3, CD20, CD43, CD30, CD31, CD34, CK20, CD79a, CDX2, myeloperoxidase, and myogenin. INI1 showed retained nuclear staining. Next‐generation sequencing revealed an *EWSR1*::*FLI* fusion, confirming the diagnosis of Ewing sarcoma. Post excisional biopsy computerized tomography (CT) scan showed diffuse rectal thickening (Figure 4) likely post‐procedural. A subsequent colonoscopy identified four polyps (tubulovillous adenoma, tubular adenoma, and hyperplastic polyp) and scar in the anus/distal rectum at the site of previous biopsy. Staging positron emission tomography (PET) scan was negative for residual/metastatic disease. Re‐excision of the previous biopsy site was negative for residual disease. The patient remained disease free for 3 years following re‐excision and is currently lost to follow‐up.

## 3 Discussion

The differential diagnosis of a poorly differentiated rectal tumor is broad and includes adenocarcinoma, squamous cell carcinoma, gastrointestinal stromal tumor, lymphoma, sarcoma, neuroendocrine lesion, and melanoma, underscoring the importance for a comprehensive immunohistochemical evaluation. In this case, the histological findings and results of immunochemical stains narrowed our considerations to dedifferentiated melanoma versus sarcoma. Initial molecular testing by PCR did not identify common BRAF or NRAS mutations. Subsequent next generation sequencing (Sarcoma Gene Fusion Panel, Mayo Clinic) revealed an *EWSR1*::*FLI1* fusion, rendering a diagnosis of Ewing sarcoma.

Ewing sarcoma is a small round cell sarcoma that frequently presents as a primary malignant bone tumor in children and young adults, frequently arising in the diaphysis or metaphysis of long bones [1]. Less commonly, Ewing sarcoma may present as an extraosseous lesion and rarely as a primary cutaneous or mucosal lesion [2, 3, 4]. Primary superficial Ewing sarcoma of the rectum is extremely rare with only one molecularly confirmed case previously reported (Table 1) [5, 6, 7].

**TABLE 1:** Summary of previously reported cases of Ewing sarcoma involving the rectum.

Case	Patient	Site	Histology	CD99	Molecular	References
1	34/F	Anal canal to rectum	Small round cells	Positive	Not performed	Aboumarzouk et al. [5]
2	17/M	Rectum	Small round somewhat nested cells	Positive	FISH: Positive *EWSR1* rearrangement, NGS: *EWSR1*::*FLI1* fusion	Drut et al. [6]
3	24/F	Rectovaginal septum to rectum	Small round cells, necrosis, scattered mitotic figures	Positive	FISH: Positive for *EWSR1* rearrangement	Ishida et al. [7]
4	53/M	Rectum	Small round blue cells, necrosis, high mitotic index	Positive	Not performed	Vardy et al. [8]

On histologic examination, conventional Ewing sarcoma consists of a monomorphic population of small round cells with minimal cytoplasm, stippled chromatin, inconspicuous nucleoli, and indistinct cell borders [2]. Tumor necrosis and hemorrhage are often present. Homer‐Wright rosettes may occasionally be identified [1]. Immunohistochemical stains are helpful in narrowing the differential diagnosis. Ewing sarcoma typically demonstrates strong and diffuse expression of CD99 (membranous staining) and NKX2.2 (nuclear staining). Additionally, reactivity for FLI1 and EGR can reflect the respective fusion partners. One potential pitfall is that CD99 reactivity may also be seen in acute lymphoblastic leukemia/lymphoma, acute myelogenous leukemia/sarcoma, small cell osteosarcoma, desmoplastic small round cell tumor (DSRCT), mesenchymal chondrosarcoma, synovial sarcoma, embryonal rhabdomyosarcoma, and *CIC*‐rearranged sarcoma, among others [1, 2]. CIC‐rearranged sarcomas exhibit similar histologic features to Ewing sarcoma as well as overlapping immunohistochemical reactivity for CD99, EGR, and INI1. Like Ewing sarcoma, CIC‐rearranged sarcomas more commonly arise in deep soft tissue; however, superficial CIC‐rearranged sarcomas have been reported [8, 9, 10, 11, 12]. While fluorescence in situ hybridization (FISH) can confirm the presence of an *EWSR1* gene rearrangement, next‐generation sequencing is able to identify both fusion partners. As previously mentioned, the *EWSR1*::*FLI1* fusion is the most common fusion (85%) followed by the *EWSR1*::*ERG* fusion (5%–10%) [11]. With the advancement and increased feasibility of performing molecular analysis, our identification and classification of fusion driven neoplasms continues to expand. For example, the *EWSR1*::*FLI1* fusion is no longer considered specific for Ewing sarcoma as the newly described superficial neurocristic *EWSR1::FLI1* fusion tumor harbors the same translocation. Superficial neurocristic *EWSR1*::*FLI1* fusion exhibits different histologic features from Ewing sarcoma and is reactive for Sox‐10 and S100, which is not characteristic of Ewing sarcoma [13]. These differences, despite identical genetic alterations, further underscore the importance for accurate histologic and immunophenotypic evaluation.

Primary superficial Ewing sarcoma is extremely rare, and this is only the second molecularly confirmed case of rectal origin. Importantly, primary superficial Ewing sarcoma has a significantly more favorable prognosis compared to its aggressive deep bone/soft tissue counterpart, which is reflected in differing treatment recommendations. While neoadjuvant chemotherapy is indicated for deeper tumors, our patient was treated only with excision and appropriate clinical follow‐up. Additionally, it is critical to distinguish Ewing sarcoma from other CD99 positive superficial sarcomas with more aggressive biologic behavior, such as CIC‐rearranged sarcoma. The discovery of tumors with identical molecular alterations, such as superficial neurocristic *EWSR1*::*FLI1* fusion tumor, reinforces the importance of interpreting molecular analysis in the context of associated histologic and immunophenotypic features.

## Conflicts of Interest

The authors declare no conflicts of interest.

## Data Availability

Data sharing not applicable to this article as no datasets were generated or analysed during the current study.
